# Genomic Insights Into the Antifungal Activity and Plant Growth-Promoting Ability in *Bacillus velezensis* CMRP 4490

**DOI:** 10.3389/fmicb.2020.618415

**Published:** 2021-01-15

**Authors:** Gustavo Manoel Teixeira, Mirela Mosela, Maria Luiza Abreu Nicoletto, Renan Augusto Ribeiro, Mariangela Hungria, Khamis Youssef, Allan Yukio Higashi, Silas Mian, André Sampaio Ferreira, Leandro Simões Azeredo Gonçalves, Ulisses de Padua Pereira, Admilton Gonçalves de Oliveira

**Affiliations:** ^1^Department of Microbiology, State University of Londrina, Londrina, Brazil; ^2^Soil Biotechnology Laboratory, Embrapa Soja, Londrina, Brazil; ^3^Agricultural Research Center, Plant Pathology Research Institute, Giza, Egypt; ^4^Department of Agronomy, State University of Londrina, Londrina, Brazil; ^5^Department of Preventive Veterinary Medicine, State University of Londrina, Londrina, Brazil; ^6^Laboratory of Electron Microscopy and Microanalysis, State University of Londrina, Londrina, Brazil

**Keywords:** root colonization ability, biosynthetic gene clusters, soilborne plant pathogens, plant-growth promoting rhizobacteria, biocontrol

## Abstract

The main objective of this study was to evaluate *Bacillus velezensis* strain CMRP 4490 regarding its ability to inhibit soil-borne plant pathogens and to increase plant growth. The study included evaluation of *in vitro* antifungal control, sequencing the bacterial genome, mining genes responsible for the synthesis of secondary metabolites, root colonization ability, and greenhouse studies for the assessment of plant growth–promoting ability. The strain was obtained from soil samples in the north of Paraná in Brazil and was classified as a *B. velezensis*, which is considered a promising biological control agent. *In vitro* assay showed that *B. velezensis* CMRP 4490 presented antagonistic activity against *Sclerotinia sclerotiorum*, *Macrophomina phaseolina*, *Botrytis cinerea*, and *Rhizoctonia solani* with a mycelial growth inhibition of approximately 60%, without any significant difference among them. To well understand this strain and to validate its effect on growth-promoting rhizobacteria, it was decided to explore its genetic content through genome sequencing, *in vitro*, and greenhouse studies. The genome of CMRP 4490 was estimated at 3,996,396 bp with a GC content of 46.4% and presents 4,042 coding DNA sequences. Biosynthetic gene clusters related to the synthesis of molecules with antifungal activity were found in the genome. Genes linked to the regulation/formation of biofilms, motility, and important properties for rhizospheric colonization were also found in the genome. Application of CMRP 4490 as a coating film on soybean increased from 55.5 to 64% on germination rates when compared to the control; no differences were observed among treatments for the maize germination. The results indicated that *B. velezensis* CMRP 4490 could be a potential biocontrol agent with plant growth–promoting ability.

## Introduction

The use of elite free-living rhizospheric microorganisms has been considered an important strategy for disease management in several crops and has earned strength in the last decade (Chen et al., 2009a; Mizuhara et al., 2011; Rückert et al., 2011; Sibponkrung et al., 2017; Liu et al., 2018). Among these soil microorganisms, the PGPR (plant growth–promoting rhizobacteria) has gained attention for their root colonization ability, high survivability, and multiplicity in root surroundings, favoring plant growth and inhibition of phytopathogens (Beneduzi et al., 2012). Based on their relationship with the plants, PGPR has been split into symbiotic and free-living; *Pseudomonas* and *Bacillus* have been widely reported as free-living microorganisms, whereas rhizobia have been considered as symbiotic plant growth promoters (Verma et al., 2019).

Different properties including the ability to form endospore, to produce a large variety of antibiotics, and to promote plant growth have recognized the genus *Bacillus* as a promising biological control agent (Podile and Kishore, 2006), being able to induce systemic defenses against diseases (Bloemberg and Lugtenberg, 2001; Beneduzi et al., 2012; Zhang et al., 2015) due to a high diversity of synthetized molecules with antimicrobial activity (Yoo et al., 2006; Rückert et al., 2011; Ji et al., 2013; Bleich et al., 2015; Zhang et al., 2015; Xu et al., 2016; Lim et al., 2017). In *Bacillus*, genes responsible for the synthesis of secondary metabolites are present in gene clusters [biosynthetic gene clusters (BGCs)] and compose a substantial part of the genome. Large-scale studies aiming to comprehend the diversity of BGCs and their roles on interaction/development of *Bacillus* suggest that conserved BGCs might be related to physiological regulations, whereas the less preserved and more diverse clusters are linked with the interaction with other organisms (Cimermancic et al., 2014; Grubbs et al., 2017). Biofilm formation has also been considered as another important property of microbial biocontrol agents (Bais et al., 2004; Fan et al., 2011).

Our objective was to evaluate the potential of *Bacillus velezensis* strain CMRP 4490 as favoring plant growth and inhibition of soil-borne plant pathogens. The study included *in vitro* evaluation of the control, sequencing the bacterial genome, mining genes responsible for the synthesis of secondary metabolites, root colonization ability, and greenhouse studies to investigate potential PGPR ability.

## Materials and Methods

### Identification of the Isolate and Phenotypic Characterization

LABIM 22 strain was originally isolated from a soil located at 23°19′26.2″S, 51°11′50.5″W, in Londrina State University (UEL), Paraná, Brazil. The strain was maintained at the Laboratory of Microbial Biotechnology at UEL and was also deposited as strain CMRP4490 at the “Coleções Microbiológicas da Rede Paranaense (CMRP)” of the Federal University of Paraná, Curitiba, Brazil. Gram staining was performed using a Gram-stain kit for morphology visualization and cell wall definition. Endospore formation was observed using the Wirtz-Conklin method. Scanning electron microscopy (SEM) was used to visualize the cell morphology and endospores.

### Complete Genome Sequencing and Assembly

For complete genome sequencing, CMRP 4490 was cultivated in LB (Luria Bertani Broth, Neogen Corporation, United States) at 150 rpm at 28°C for 48 h. DNA extraction was obtained with the Gentra Puregene Genomic DNA kit, Qiagen Brazil, according to the manufacturer’s procedure, and quantified. Sequencing was carried on the Illumina MiSeq platform, using a MiSeq version 3 reagent kit (600-cycle, Illumina, Brazil) at the Soil Biotechnology Laboratory in Embrapa Soja, Londrina, Paraná, Brazil. The reads’ quality and the trimming parameters were observed and chosen using FastQC, setting a threshold Phred score of 30, with several trimming parameters to obtain the best data possible for *de novo* assembly (Leggett et al., 2013; Bolger et al., 2014). A series of *de novo* assemblies were carried out with different software (SPAdes, Velvet, IDBA hybrid and CLC Genomic Workbench 11) (Zerbino and Birney, 2008; Peng et al., 2010), and the assemblies were compared with QUAST (Gurevich et al., 2013). Metrics including total alignment size, number of contigs, biggest contig, N50 values, and numbers of genes according to the reference genome provided on QUAST were used to choose the best assembly. Contigs were aligned with a reference genome using the webserver CONTIGuator (Galardini et al., 2011), and raw reads from the sequencing were mapped against the scaffolds generated (Langmead and Salzberg, 2012), and the ones with low read count to support the sequence were discarded. Gaps within the scaffold were first treated with the module GapCloser (Luo et al., 2012) followed by manual curation with reading mapping using Bowtie2 and gap-filling using CLC Genomics Workbench 11 GUI (Qiagen, United States). Genome start was determined by comparison with the reference strain *B. velezensis* Bac57 considering the gene dnaA as the first gene. Genome annotation was realized using the RAST platform (Aziz et al., 2008), and coding sequence (CDS) were predicted and classified into subsystems.

### Representation of Circularized Genome and Secondary Metabolites Cluster Prediction

The genome of strain CMRP 4490 was represented circularly and compared with other reference genomes using the BRIG (BLAST Ring Image Generator) software (Alikhan et al., 2011). The webserver antiSMASH combines different databases of genetic data, antimicrobial molecules, and BGCs to predict the position and possible function of the clusters (Blin et al., 2019). The analysis was carried out with the final fasta file of CMRP 4490.

### Phylogenomic Comparison and Tree

For species determination, ANI (Average nucleotide Identity) and dDDH (digital DNA-DNA hybridization) between other *Bacillus* spp. were determined using orthoANI (Lee et al., 2016) and Genome-to-Genome Distance Calculator (GGDC) (Meier-Kolthoff et al., 2013), respectively. Using the Gegenees software, whole-genome comparisons were made with CMRP 4490 and other closely related strains (Agren et al., 2012). Data from Gegenees were exported to SplitsTree for tree confection using the UPGMA method (Legendre and Legendre, 2012).

### *In vitro* Antifungal Assays

For the antifungal activity analysis, dual culture assays were carried out. For the strain’s activation, CMRP 4490 was cultured in LBA (Luria Bertani Agar, Neogen Corporation, United States) at 28°C for 24 h. Then, CMRP 4490 was inoculated, using an inoculation loop, at four points at the edges of plates containing PDA medium (Potato Dextrose Agar, Neogen Corporation, United States). On the center of the Petri dish, a 6-mm mycelial plug taken from the edge of actively growing colonies of three phytopathogenic fungi (*Sclerotinia sclerotiorum*, *Macrophomina phaseolina*, *Botrytis cinerea*, and *Rhizoctonia solani*) was placed. The experiment was incubated at 25°C with a 12-h/12-h photoperiod for 7 days. For comparison purposes, positive control was performed with only the mycelial disc at the center of the Petri dish incubated under the same conditions. Growth was calculated as the average of the two orthogonal diameters (mm), and growth inhibition was determined using the following formula:

(1) $$\mathrm{MGI}\%=\left[\frac{\mathrm{cd}-\mathrm{td}}{\mathrm{cd}}\right]\times 100$$

where cd = diameter in control, td = diameter in treatment, and MGI = mycelial growth inhibition.

### Biofilm Formation Related Genes

After genome annotation, a local BLASTN using a customized database containing the CMRP 4490 genome was made using as query sequences a list of genes directly or indirectly related to biofilm formation/regulation found on SubtWiki and other articles (Robertson et al., 1989; Strauch et al., 1990, 2007; Msadek et al., 1991; Kobayashi, 2007; Chu et al., 2008; Penha et al., 2020) related to biofilms of *Bacillus subtilis.*

### Swarming Motility and Pellicle Formation Assay

This ability was evaluated by spreading the bacterium on tryptic soy agar plates 0,85% (TSA, Acumedia, United States). An overnight culture was prepared and adjusted to 0.5 on the McFarland scale (1.5 × 10^8^ CFU mL^–1^), and 10 μL of this inoculum was placed in the middle of a 60-mm Petri dish. The ability to colonize the whole surface of the plate was evaluated. For pellicle formation, a 24-well plate with 2 mL of tryptic soy broth medium (Acumedia, United States) was prepared and inoculated with 10 μL of an overnight culture adjusted to 0.5 on the McFarland scale. Digital pictures were taken to evaluate the pellicle formation capacity.

### Root Colonization Capacity With SEM

To analyze the CMRP 4490 colonization capacity, maize and soybean seeds were surface-disinfected by immersion in 95% (vol/vol) ethanol solution for 30 s, followed by soaking in 0.5% NaOCl for 10 min, and washed six times with sterile deionized water. The seeds were treated with lyophilized cell cultures at three different concentrations (100, 200, and 300 g of lyophilized cell culture for 100 kg of seeds). The commercial product Presence^®^ (FMC Corporation containing *B. subtilis* strain FMCH002 and *Bacillus licheniformis* strain FMCH001) was used as a positive control (200 g for 100 kg of seeds). Negative control with non-treated surface-disinfected seeds was included. The seeds were treated in a Becker (600 mL for 100 kg of seeds), followed by agitation for homogenization for 1 min. Seeds were incubated for 7 days on a germination paper moistened with sterilized distilled water and incubated in a growth chamber at 25°C with 70% RH. On day 0, four seeds of each treatment were picked for sample preparation for SEM analysis. After 7 days, four germinated seeds were used from each treatment for sample preparation focusing on the radicular area. Samples were fixed with glutaraldehyde 2.5% and sodium cacodylate buffer at 0.1 M for 24 h and then dehydrated on increasing alcohol concentrations for 15 min at each concentration (30, 50, 70, 90, and 100%). Finally, the samples were critical-point-dried with CO_2_ (BALTEC CPD 030 Critical Point Dryer), attached to a metal stub and covered with gold (BALTEC SDC 050 Sputter Coater) for SEM visualization (FEI Quanta 200, Netherlands).

### Germination and Growth Promotion on Zea Mays and Glycine Max

The following treatments were evaluated: T1: negative control (without application of the cells); T2: commercial product Presence^®^ (200 g for 100 kg of seeds); and T3, T4, and T5: *B. velezensis* strain CMRP 4490 (100, 200, and 300 g, respectively, of lyophilized cell culture for 100 kg of seeds). The maize and soybean cultivars used were BALU 388 VIP3, and BMX Potência RR, respectively. For the preparation of lyophilized cells, strain CMRP 4490 was cultivated for preinoculum preparation on a saline solution (0.85% sodium chloride) and adjusted to 0.5 on the MacFarland scale. For inoculum preparation, 30 μL of this solution was added to an Erlenmeyer flask of 125 mL containing 30 mL of LB broth and incubated at 28°C for 24 h at 125 rpm (Orbital Shaker Thoth 6430, Brazil). For fermentation, 4 μL of the inoculum was added to 400 mL of formulated medium (patent applications BR 10 2020 013481 7) on 1,000 mL Erlenmeyer flasks and incubated for 72 h at 28°C and 200 rpm. The content of the flasks was frozen at −80°C and lyophilized at −60°C to obtain a powder containing CMRP 4490 metabolites and spores at 1 × 10^10^ CFU/g.

For the germination and growth-promoting experiments, the seeds were initially disinfected by immersion in 95% (vol/vol) ethanol solution for 30 s, soaked in 0.5% NaOCl for 10 min, and then washed six times with sterile deionized water. In a beaker, the seeds were treated with three different concentrations of lyophilized cells following the recommended of 600 mL for 100 kg of seeds, and the Becker was agitated manually for 1 min for homogenization.

To evaluate the germination, 50 seeds per treatment were placed on germination paper moistened with sterilized distilled water and incubated in a growth chamber at 25°C ± 2°C and 70% relative humidity for 7 days. A completely randomized statistical design with four replicates was used.

To evaluate the growth promoting, the treated seeds were spread on 900 mL Styrofoam pots with sand, leaving three plants for each pot. Fifteen days after sowing, the seedlings were scanned at 300 dpi, and the images were treated and analyzed with the software GiARoots (Galkovskyi et al., 2012). The total root surface (cm^3^) and total root length (mm) were evaluated. Then, seedling shoots and roots were separated and oven-dried separately under forced ventilation at 60°C for 72 h to determine shoot and root dry weight (g). For the experiment on growth promotion, a completely randomized design with six replicates was used.

All data were submitted to analysis of variance (ANOVA), and means were compared using Tukey test (*p* ≤ 0.05). All data were analyzed using R^1^ with the agricolae package (De Mendiburu and Simon, 2015).

## Results

### Phenotypical Characterization

Gram staining showed that CMRP 4490 is a Gram-positive rod-shaped bacterium and produces endospores positioned at the terminal part of the cellule. The rod-shaped morphology, cell association, and the endospore were shown by SEM (Supplementary Figure 1).

### Genome Assembly and Annotation

Following the proposed strategy, CLC Genomics Workbench 11 and IDBA Hybrid have shown the best results concerning assembly metrics. A BLASTN was carried out using the biggest contig to find a reference genome for the CONTIGuator step. The strain Bac57 of *B. velezensis* (NZ_CP033054.1) was selected to align the contigs and generate the pseudocontig (scaffold). The scaffold contained 64 gaps that were primarily treated with GapCloser (Kremer et al., 2017) and then manually curated using Bowtie2 and CLC Genomics Workbench 11. When closed, the genome showed a 99.03% rate of alignment of the reads, a size of 3,996.396 bp. Genome annotation showed that the genome possesses a GC content of 46.4%, 4,042 CDS being 21 of those related to rRNA and 85 tRNA sequences. The sequence was deposited at the GenBank and received the accession number CP045993 (Table 1).

**TABLE 1:** Features of *B. velezensis* CMRP 4490 genome.

**Genome size (bp)**	**3,996,396**
GC content (%)	46.4
Plasmid	1
Total predicted CDS	4,042
rRNA operons	21
tRNA operons	85
Read alignment	99.03%
Access genome n° GenBank	CP045993
Access plasmid n° GenBank	CP061026

### Strain Identification and Phylogenomic Analysis

**FIGURE 1:**
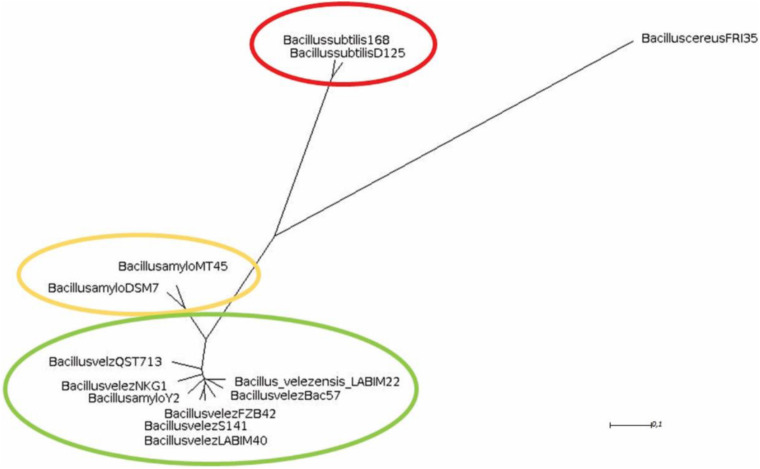
Phylogenomic tree representing the similarity between strains from the *B. subtilis* group. The matrix was generated by Gegenees and exported to SplitsTree. The most similar strains to CMRP 4490 belonging to the *B. velezensis* species were highlighted with green color. In yellow are the *B. amyloliquefaciens* species, less similar to CMRP 4490, and highlighted in red are the *B. subtilis* species with a *B. cereus* FRI35 as an extern group.

When compared to several strains, including type strains, of three main species from the *B. subtilis* group, the ANI and dDDH indicated that closer similarity of strain CMRP 4490 went with *B. velezensis* Bac57, of 99.26 and 94.30%, respectively (Table 2). Other strains showing high similarity with CMRP 4490 (ANI, and dDDH, respectively) were *B. velezensis* S141 (98.24%, 84.90%), *B. velezensis* NKG-1 (98.19%, 84.70%), *B. velezensis* type strain FZB42 (98.23%, 85.70%), and *B. velezensis* QST713 (98.30%, 85.10%), above the threshold for species delineation. When compared to *B. amyloliquefaciens* type strain DSM7(T), the values of 93.67% for ANI and 56% for dDDH indicate that CMRP 4490 and type strain DSM7(T) belong to different species; this classification is important for this group because of the inconsistent delimitation over the years (Kim et al., 2015; Dunlap et al., 2016; Fan et al., 2017). A lower similarity of CMRP 4490 was shown with *B. subtilis* D12-5 and *B. subtilis* subsp. *subtilis* type strain 168 (80.36%, 20.80%, and 80.36%, 20.90%, respectively). Because of the comparisons made with Gegenees and orthoANI/GGDC, CMRP 4490 is located within a cluster containing most of the *B. velezensis* species (Figure 1).

**TABLE 2:** Genome comparison of CMRP 4490 with other *Bacillus* species.

**Strains**	**Accession n° on GenBank**	**dDDH%**	**ANI (%)**	**GC (%)**	**Bp (Gb)**	**Source**
***B. velezensis***						
**CMRP 4490**	**CP045993**	**100**	**100**	**46.40**	**3.99**	**Soil**
S141	AP018402.1	84.90	98.24	46.50	4.03	Soybean rhizosphere (Sibponkrung et al., 2017)
NKG-1	CP024203.1	84.70	98.19	46.30	4.19	Volcanic soils at the Changbai mountains (Ge et al., 2016)
Bac57	CP033054.1	94.30	99.26	45.89	4.23	Red Sea lagoon
LABIM40	CP025079.1	88.10	98.22	46.50	3.97	Contaminant (Baptista et al., 2018)
FZB42	CP000560.2	85.70	98.23	46.50	3.91	Soil (Chen et al., 2009a)
QST713	CP025079	85.10	98,30	45.90	4.23	Commercial product Bayer^®^ (Pandin et al., 2010)
***B. amyloliquefaciens***						
Y2	NC_017912.1	92.90	99.15	45.90	4.23	Wheat Rhizosphere (Hao et al., 2012)
DSM7(T)	NC_014551.1	56.00	93.67	46.10	3.98	Not specified (Rückert et al., 2011)
MT45	CP011252.1	56.00	94,13	46.10	3.89	Not specified
***B. subtilis***						
D125	CP020102.1	20.80	80.36	43.60	4.14	Soil (Niazi et al., 2014)
168	NC_000964.3	20.90	80.36	43.50	4.21	Type strain (Kunst et al., 1997)
***B. cereus***						
FRI-35	CP003747.1	39.30	68.10	35.45	5.38	Not specified

*dDDH, digital DNA-DNA hybridization; ANI, average nucleotide identity, GC, content of guanine and cytosine on the genome; Bp, genome size in gigabases (10^6^).*

### antiSMASH Analysis of Secondary Metabolism

**FIGURE 2:**
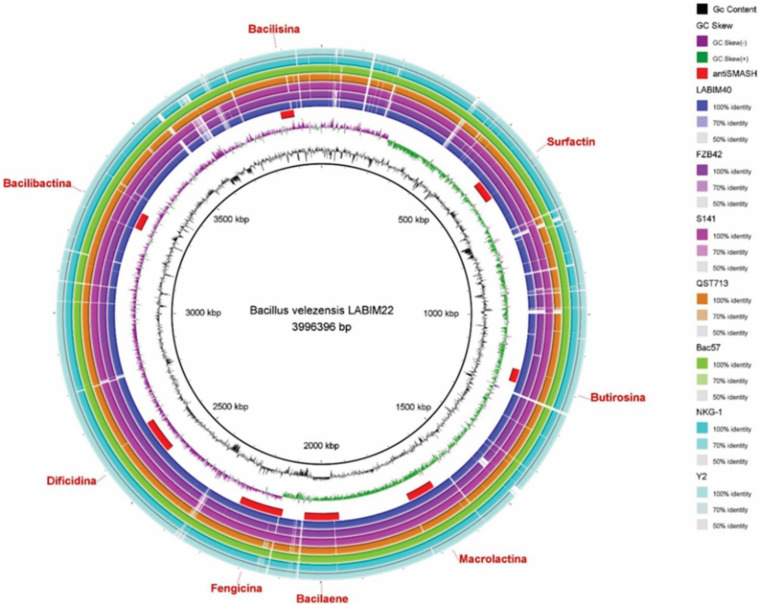
Circular representation of the genome of *B. velezensis* strain CMRP 4490 using the program BRIG. From the inside to the outside the legends are as follows: (Mizuhara et al., 2011) GC content, (Chen et al., 2009a) GC skew, (Sibponkrung et al., 2017) BGCs position on the genome indicated by antiSMASH (Bloemberg and Lugtenberg, 2001; Podile and Kishore, 2006; Rückert et al., 2011; Beneduzi et al., 2012; Zhang et al., 2015; Liu et al., 2018; Verma et al., 2019), respectively, LABIM40, FZB42, S141, QST713, Bac57, NKG-1, and Y2.

The webserver antiSMASH 5.1.0 found 13 BGCs on the CMRP 4490 genome (Table 3). Clusters were also represented in the genome circular image (Figure 2). Among the 13 clusters, 8 showed similarity with BGCs already described on the MiBiG (Medema et al., 2015). They were linked to the synthesis of surfactin, butirosin A/B, macrolactin H, bacillaene, fengycin, difficidin, bacillibactin, and bacilysin. Five of the 13 clusters did not show any similarity clusters in the database.

**TABLE 3:** Biosynthetic Gene Clusters (BGCs) found within CMRP 4490 genome using the webserver antiSMASH 5.1.0.

**Cluster**	**Type**	**From (pb)**	**To (pb)**	**Most similar known cluster**		**Similarity**
1	NRPS	556,245	620,583	Surfactin	NRP: lipopeptide	82%
2	Phosphonate	855,967	896,851			
3	PKS-like	1,173,665	1,214,909	Butirosin A/butirosin B	Saccharide	7%
4	Terpene	1,307,518	1,324,794			
5	TransAT-PKS	1,631,362	1,717,721	Macrolactin H	Polyketide	100%
6	TransAT-PKS, T3PKS, TransAT-PKS-like, NRPS	1,943,834	2,052,707	Bacillaene	Polyketide + NRP	100%
7	NRPS, transAT-PKS, beta-lactone	2,123,165	2,259,899	Fengycin	NRP	100%
8	terpene	2,283,957	2,305,840			
9	T3PKS	2,381,461	2,422,561			
10	TransAT-PKS-like, transAT-PKS	2,537,196	2,643,371	Difficidin	Polyketide + NRP	100%
11	NRPS, bacteriocin	3,272,003	3,322,513	Bacillibactin	NRP	100%
12	NRPS	3,599,816	3,665,158			
13	other	3,867,578	3,908,996	Bacilysin	Other	100%

*NRPS, non-ribosomal peptide synthetase; NRP, non-ribosomal peptide. PKS, polyketide synthetase. AT, acetyltransferase; T3PKS, type 3 Pks.*

### *In vitro* Antifungal Assays

**FIGURE 3:**
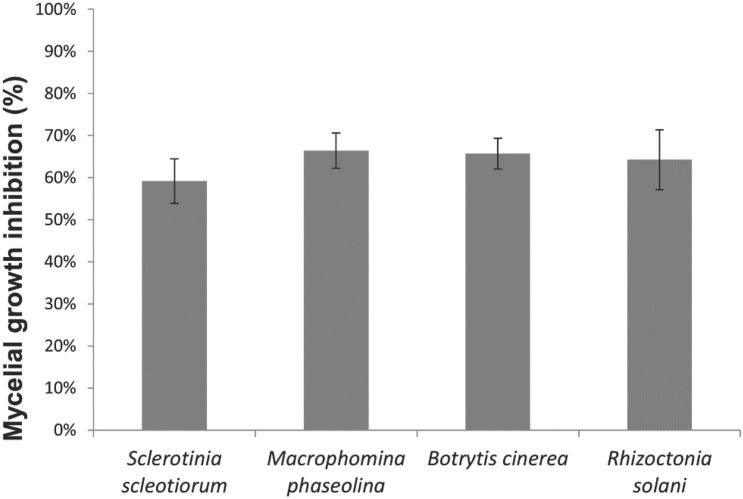
Mycelial growth inhibition (%) for *S. sclerotiorum*, *M. phaseolina*, *B. cinerea*, and *R. solani* by *B. velezensis* strain CMRP 4490. The data are represented by the gray bar graph with their respective standard deviation values.

The direct antagonistic activity of strain CMRP 4490 is shown in Figure 3. The strain showed antifungal activity against *S. sclerotiorum*, *M. phaseolina*, *B. cinerea*, and *R. solani* with an MGI of approximately 60%, without any significant difference among them. No contact is observed between the two microorganisms, indicating a clear antagonistic effect by CMRP 4490.

### Genes Related to Biofilm Formation/Regulation and *in vitro* Assays

**FIGURE 4:**
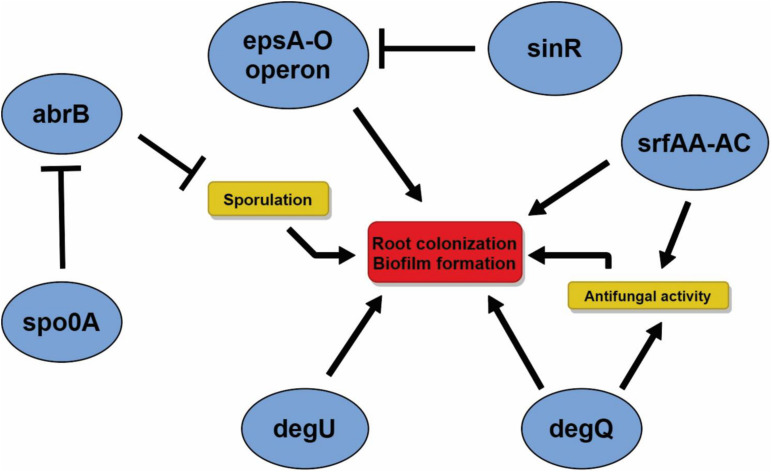
Representation of the main genes found in the genome of *B. velezensis* strain CMRP 4490 related to the antifungal activity and sporulation inducing the biofilm formation/regulation, properties impacting root colonization. Arrows indicate positive regulation, and “T” bars indicate repression of the gene expression.

A series of genes directly or indirectly related to the biofilm production and root colonization were found on the CMRP 4490 genome (Supplementary Table 1). Key genes such as spo0A (99% of similarity with genes obtained on the SubtWiki database), abrB (91%), sinR (97%), degU (86%), and degQ (87%) were found within the genome using BLASTN. Important operons, such as epsA-O (∼99%), sfrAA-AC (79%, 75%, 87%) and yqxX-sipW-tasA (99%, 99%, 99%), were also found on the chromosome sequence. *In vitro* assays showed that CMRP 4490 was competent at biofilm formation, and its motility capacity and pellicle formation ability are shown in Supplementary Figure 2. For the motility test, it was observed that the cell inoculum starting in the middle of the Petri dish was able to grow over the whole surface of the TSA 0,8% medium (Supplementary Figure 2A). Pellicle formation on the air–liquid interface is visible in Supplementary Figure 2B, where a thick layer, resulting from the cell growth, covers the whole air–liquid surface on the well. With these results, we had an insight into the interaction of genes related to root colonization and biofilm formation, and those relations are important for future studies aiming to genetically regulate CMRP 4490 root colonization capacity through genes associated with this pathway (Figure 4).

### Root Colonization on *in vivo* Assays

**FIGURE 5:**
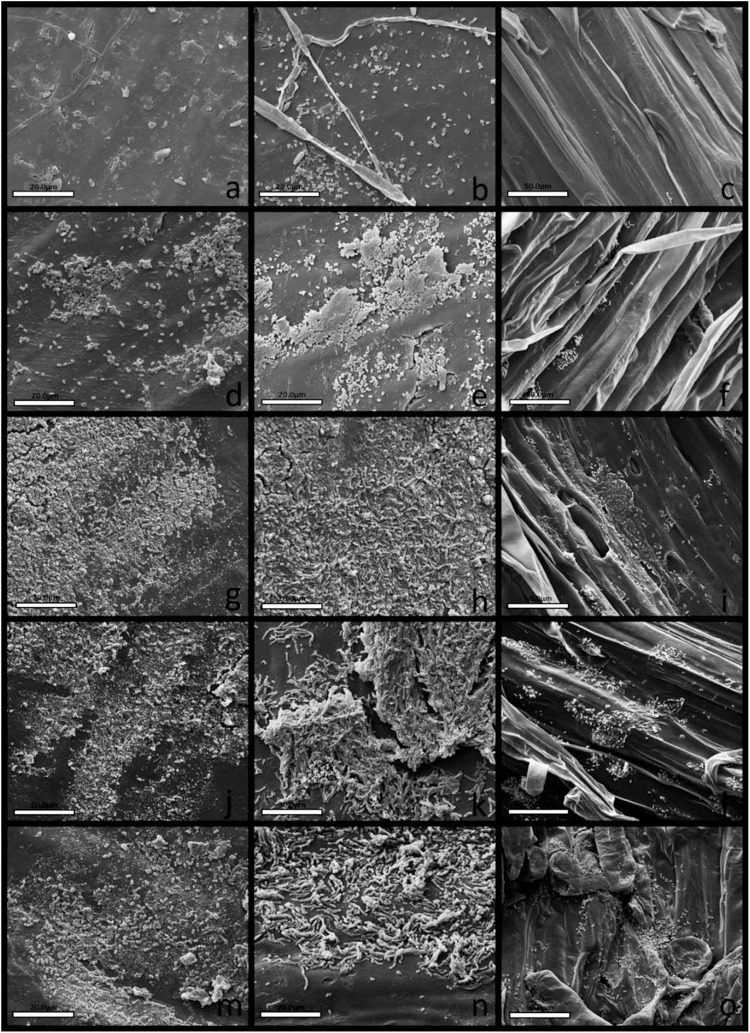
Scanning electron microscopy of maize seeds’ surfaces and roots colonization by *B. velezensis* strain CMRP 4490. The first column shows the seeds’ surfaces on all treatments on the first day (3,000×), the second column shows the seeds’ surfaces after 7 days, the third column shows roots amplifications after 7 days (2,000×). **(a–c)** Negative control without biological products. **(d–f)** Positive control treated with Presence^®^ (200 g/100 kg of seeds). **(g–i)** Treatment with *B. velezensis* strain CMRP 4490 lyophilized (100 g/100 kg of seeds). **(j–l)** Treatment with *B. velezensis* strain CMRP 4490 lyophilized (200 g/100 kg of seeds). **(m–o)** Treatment with *B. velezensis* strain CMRP 4490 lyophilized (300 g/100 kg of seeds). All treatments with *B. velezensis* strain CMRP 4490 lyophilized **(g,j,m)** exhibited cells attached to seed surfaces and exhibited successfully maize root colonization **(h,k,n)**. The *B. velezensis* strain CMRP 4490 concentration of viable spores per gram of lyophile was 1 × 10^10^ CFU/g.

**FIGURE 6:**
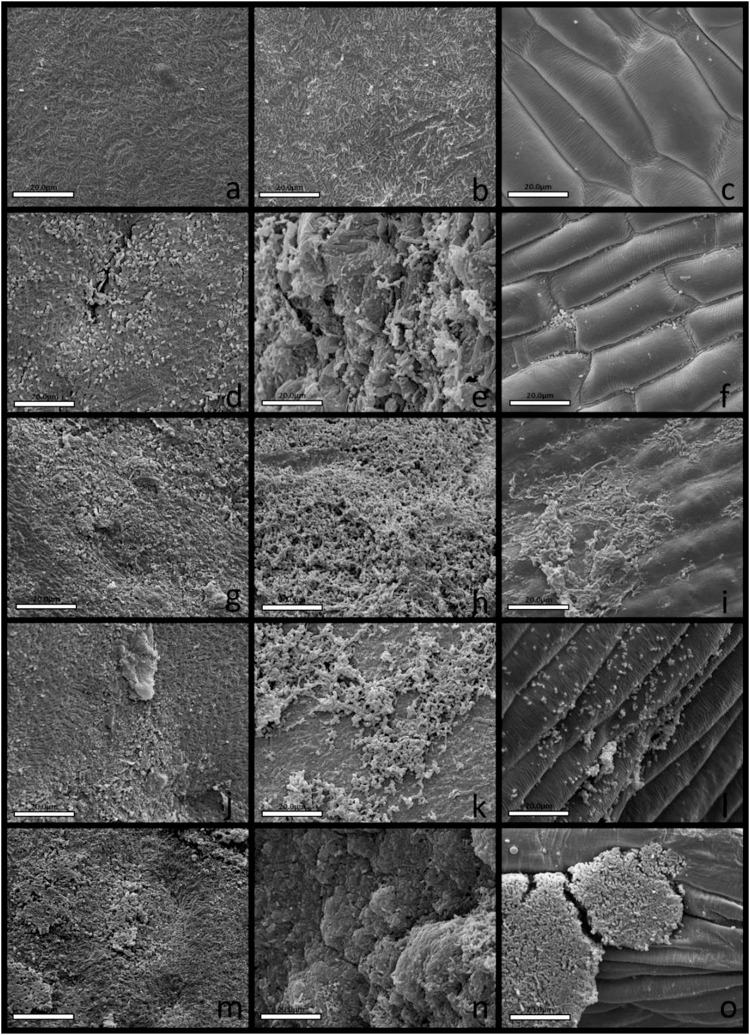
Scanning electron microscopy of soybean seeds’ surfaces and roots colonization by *B. velezensis* strain CMRP 4490. The first column shows the seeds’ surfaces on all treatments on the first day (3,000×), the second column shows the seeds’ surfaces after 7 days, the third column shows roots amplifications after 7 days (2,000×). **(a–c)** Negative control without biological products. **(d–f)** Positive control treated with Presence^®^ (200 g/100 kg of seeds). **(g–i)** Treatment with *B. velezensis* strain CMRP 4490 lyophilized (100 g/100 kg of seeds). **(j–l)** Treatment with *B. velezensis* strain CMRP 4490 lyophilized (200 g/100 kg of seeds). **(m–o)** Treatment with *B. velezensis* strain CMRP 4490 lyophilized (300 g/100 kg of seeds). All treatments with *B. velezensis* strain CMRP 4490 lyophilized **(g,j,m)** exhibited cells attached to seeds’ surfaces and exhibited successfully maize roots colonization **(h,k,n)**. The *B. velezensis* strain CMRP 4490 concentration of viable spores per gram of lyophile was 1 × 10^10^ CFU/g.

Strain CMRP 4490 was able to attach and multiply on the seed surface of both maize and soybean (Figures 5, 6), important features for the introduction of the bacteria into the rhizosphere (Danhorn and Fuqua, 2007), as well as being able to colonize the initial root formation on the seedlings. The first column indicates seeds on the first day of treatment, the second shows the seeds after 7 days of growth, and the third shows the initial root formation from the seedlings. On both figures from A to C T1 is shown, from D to F are T2 with the commercial biological product Presence^®^ at 200 g/100 kg of seeds, from G to I the first treatment with CMRP 4490 cells at 100 g/100 kg of seeds (T3), from J to L the second treatment with 200 g/100 kg (T4), and from M to O the third treatment with 300 g/100 kg (T5). With the three concentrations tested, CMRP 4490 colonized the root at various parts of the tissue and formed cell aggregates. A primary biofilm formation can be spotted at items I and L in Figure 5 covering a group of cells attached to the root. The results corroborate with data found on CMRP 4490 genome and the *in vitro* assays proving its mobility and biofilm formation capacity.

### Germination and Growth-Promoting Ability

Based on the ANOVA, no differences were observed among treatments for the maize germination parameter but were confirmed for soybean. Soybean germination rates increased with CMRP 4490 treatments (from 55.5 to 64%) when compared to the control (44%) (Table 4). No significant difference between treatments was detected concerning the root and shoot dry weight. However, for the radicular properties, the CMRP 4490 treatments resulted in differences in total root length on both maize and soybean and total root surface area in soybean (Table 4). Total root length in soybean was increased in all treatments with CMRP 4490 when compared to both negative and positive controls, whereas maize plants increased total root length only with the highest concentration of CMRP 4490 (Table 4). For soybeans, all concentrations increased total root length, the total surface area increased on all treatments showing no difference between the CMRP 4490 treatments and the positive control (Table 4).

**TABLE 4:** Effect of *B. velezensis* strain CMRP 4490 inoculation on traits related to germination and growth-promoting in maize and soybean crops.

**Treatments**	**Variables^1^—maize**				
	**GERM (%)**	**RDW**	**SDW**	**TRS**	**TRL**
Control	94.00	0.3155	0.1883	294.75	213.49b
Presence (200g/100 kg)	97.00	0.3748	0.1851	292.72	217.88b
CMRP 4490 (100 g/100 kg)	97.50	0.3221	0.1834	296.61	214.26b
CMRP 4490 (200 g/100 kg)	99.00	0.3218	0.2137	294.66	218.57b
CMRP 4490 (300 g/100 kg)	97.5	0.3287	0.2068	318.92	252.65a
**Treatments**	**Variables^1^—soybean**				
	**GERM (%)**	**RDW**	**SDW**	**TRS**	**TRL**

Control	44.00b	0.1203	0.1706	255.27b	145.21b
Presence (200g/100 kg)	61.50a	0.1225	0.2106	280.13*ab*	158.58b
CMRP 4490 (100 g/100 kg)	64.00a	0.1025	0.1807	324.37a	189.79a
CMRP 4490 (200 g/100 kg)	63.00a	0.1125	0.1862	323.31a	192.86a
CMRP 4490 (300 g/100 kg)	55.50*ab*	0.1115	0.1810	287.93*ab*	193.19a

*GERM, germination in percentage; RDW, root dry weight (g); SDW, shoot dry weight (g); TRS, total root surface (cm^3^); and TRL, total root length (mm). ^1^Means followed by the same letter in the column do not differ by the Tukey test, at a significance level of 5% of probability.*

## Discussion

*Bacillus velezensis* CMRP 4490 (LABIM 22) strain was originally isolated from a soil located in Londrina State University (UEL), Paraná, Brazil. In preliminary studies, CMRP 4490 showed a potent antagonism against *Fusarium solani* 145, *F. solani* 234, and *Fusarium oxysporum* f. sp. *phaseoli* 06 (Higashi, 2020). To determine which are the best conditions for fermentation and production of bioactive by the LABIM22 strain, we studied the best culture medium for the production of antifungal metabolites (patent applications BR 10 2020 013481 7). Aiming to reveal its potential as a biocontrol agent against important phytopathogenic fungi, we investigated the genomic and physiological properties of *B. velezensis* strain CMRP 4490. More specifically, we sequenced its genome, investigated genes of the secondary metabolism, mining for genes linked to biofilm regulation/formation, and testing its plant growth–promoting ability.

Genomic analysis has revealed that *B. velezensis* CMRP 4490 possesses specific clusters of genes related to the biosynthesis of secondary metabolites, which play significant roles in both pathogen suppression and plant growth promotion, and to genes related to rhizospheric colonization. Other studies also describe this potential for strains of *B. velezensis;* as an example, S141 isolate and the reference *B. velezensis* FZB42 showed the high capacity of growth promotion, mainly on soybean (Chen et al., 2009a; Sibponkrung et al., 2017), suggesting the presence of such a capacity within the CMRP 4490 strain due to the high genomic similarity. In another study, *B. velezensis* NKG-1 showed antifungal activity *in vitro* tests, showing antagonism against 13 phytopathogenic fungi (Ge et al., 2016; Liu et al., 2018); its high similarity with CMRP 4490 draws attention to a possible wider range of antifungal activity against fungi not investigated yet. *B. subtilis* 168 is a model bacterium for biofilm studies, although less similar to CMRP 4490 than B. *amyloliquefaciens*, its genomic resemblance with CMRP 4490 suggests a potential biofilm formation capacity contained in the genome. All these comparisons made with closely related *Bacillus* spp. suggested that CMRP 4490 possesses different potentials in its genome that make the strain a good candidate as a biocontrol agent.

Among the great diversity of molecules produced by *Bacillus* spp., lipopeptides are known for showing antimicrobial activity (Simon et al., 2019). This group of molecules has also been related to root colonization capacity and cell motility when present on the extracellular matrix (Kinsinger et al., 2003; Grubbs et al., 2017), attributed to its capacity of decreasing the superficial tension; besides, surfactins have been related to the induction of systemic resistance by the plant (Ongena et al., 2007). Also, surfactin can induce systemic resistance by the plant against potential phytopathogens (Ongena et al., 2007). This cluster is important because it might be directly related to the antifungal activity shown by CMRP 4490 on the *in vitro* assays against phytopathogenic fungi, possibly being the target of future *in silico* studies aiming at its regulatory pathways. Macrolactins represent a group of macrolides found in deep-sea bacteria and have also shown antimicrobial activity (Lu et al., 2008; Sohn et al., 2008) and growth inhibition of melanomas (Gustafson et al., 1989). The seventh cluster found in CMRP 4490 contains all genes related to the biosynthesis of fengycin, a lipopeptide with an antifungal activity that alters the membrane permeability of the target cell causing its death (Vanittanakom et al., 1986; Deleu et al., 2008).

Other studies performed with the reference strain *B. velezensis* FZB42 associated the antagonistic activity against *Erwinia amylovora*, the causal agent of fire blight on apples, pears, and some other members of the family Rosaceae (Myung et al., 2016), with the production of difficidin and bacilysin (Chen et al., 2009b). The results obtained herein indicated that two clusters found in the genome of CMRP 4490 genome showed 100% similarity to the database MiBiG of the synthesis of these molecules, with potential against bacterial diseases not explored yet. The 13th cluster showed 100% similarity with a cluster of bacillibactin production, an important siderophore (Dertz et al., 2006; Miethke et al., 2006). *Bacillus* species able to produce siderophores already have been shown to present antifungal activity (Yu et al., 2011). Five of the 13 clusters identified in strain CMRP 4490 did not show any similarity in the database; therefore, their products are yet to be identified and described, opening opportunities for further studies. Thus, we detected a great quantity of gene clusters relating to synthesis of antimicrobial metabolites, which contained various enzyme-encoding operons for non-ribosomal peptide synthetases and polyketide synthases, as well as clusters with a potential for novelty. These results indicate that *B. velezensis* strain CMRP 4490 could be a potential biological agent to protect plants. In addition to the results obtained in *in silico* analyzes, *B. velezensis* strain CMRP 4490 showed a great antagonism *in vitro* against *S. sclerotiorum*, *M. phaseolina*, *B. cinerea*, and *R. solani*. These soil-borne fungi are diverse and difficult to manage in agriculture. Therefore, it is extremely important to develop techniques or products for handling these soil-borne fungal pathogens that cause widespread damage, reducing the yield of many economically important crops. The findings obtained by this study are consistent with other studies for *B. velezensis* against phytopathogenic fungi (Ge et al., 2016; Xu et al., 2016; Lim et al., 2017).

Another important attribute evaluated in the *B. velezensis* strain CMRP 4490 genome, very important for the colonization process, were the genes directly or indirectly related to the formation/regulation of biofilm. Some have been detected, such as abrB, a gene repressor that induces the endospore formation (Robertson et al., 1989; Strauch et al., 2007), considered as a key cell physiological state for the formation of complex biofilms. The gene abrB interacts with spoOA, another gene found in the CMRP 4490 genome and whose product has shown an affinity for the promoting region of abrB (Strauch et al., 1990); its putative role as regulator represents an interesting target for regulation strategies. Another gene, degQ has its expression associated with antifungal activity, and mutation implies low production of lipopeptides, the main group of molecules related to the antifungal activity on *Bacillus* spp. (Ongena and Jacques, 2008). The gene degU was also found in CMRP 4490, and its mutation indicated a putative role in biofilm formation or regulation (Strauch et al., 2007). A repressor found on the genome was sinR, a gene with affinity with the promoting region of the epsA-O operon (Kearns et al., 2004), related to the synthesis of exopolysaccharides, important for biofilm settling on the substrate (Vlamakis et al., 2013). All genes from this operon were present in the genome of CMRP 4490. Another important operon detected was swrA-C, responsible for the swarm that helps bacterial motility, an important property for root colonization. Surfactins support the swarming by decreasing the superficial tension on the extracellular matrix from the biofilm allowing larger mobility to the cells composing the biofilm (Kearns and Losick, 2004). Our results of SEM analysis provided useful information about the root colonization capacity of strain CMRP 4490. The results obtained suggest that *B. velezensis* CMRP 4490 can also be used as a biocontrol agent applied to the seeds, as it is capable of colonizing the seed surface and can continue its life cycle until initial root formation. The strain is also capable of colonizing and form biofilms (Schroth and Hancock, 1982; Backer et al., 2018). Besides, the motility capacity shown in *in vitro* assays and the presence of important genes related to the motility capacity CMRP 4490 suggest that the genes related to these properties found in the genome mining are functional and give the strains the ability to colonize roots (de Weert et al., 2002; Kinsinger et al., 2003). Hence, direct seed coating seems to have beneficial effects on seed germination. This could be due to the ability of the selected strain to produce auxin-like phytohormones. Many reports are showing that *Bacillus* spp. can enhance plant growth by producing different plant growth hormones such as gibberellins, indole acetic acid, and cytokinins (Arkhipova et al., 2005; Radhakrishnan and Lee, 2016). It has been also reported that some *Bacillus* spp. can produce more than one phytohormone, with beneficial effects on plants. Further analyses are still needed to better comprehend the positive biological effect of CMRP 4490 on germination promotion. The increase in total root length induced by CMRP 4490 on maize roots was achieved only with the highest concentrations, suggesting that higher doses are needed. Besides, the increment of total root length on soybean crops with all CMRP 4490 treatments highlights its potential as a root growth inducer for this crop. Analyzing the soybean root total surface area, it is noted that the higher product concentration slightly decreased root growth, a common response to excess of phytohormones. The obtained results support the usage of CMRP 4490 based products as a PGPR mainly for root properties.

## Conclusion

With the presented work, it was possible to affirm that *B. velezensis* CMRP 4490 harbors a biotechnological potential because of its high genetic similarity with other strains from the *Bacillus* genus already used as biocontrol control agents and already established on the market. This potential is reinforced by the presence of genes found in the genome that were directly and indirectly associated with biofilm formation/regulation and by the exhibited capacity of colonizing roots *in vivo*. The study of the genetic machinery linked with the secondary metabolism also strengthens the biotechnological potential of this strain by finding BGCs that are related to the antifungal activity. Besides, BGCs did not have any similarity with others described on the database that can be targets of *in silico* studies focusing on the diversity of molecules that can be synthetized by CMRP 4490 during its secondary metabolism. Finally, its growth-promoting capacity described in this work classifies CMRP 4490 as a PGPR, an important characteristic of a bacterium that is aimed to be used as a potential plant growth–promoting agent.

## Accession Numbers

The whole genome is deposited at the DDBJ/EMBL/GenBank. BioProject (PRJNA588786), BioSample (SAMN13256649), and Accession (CP045993).

## Data Availability Statement

The datasets presented in this study can be found in online repositories. The names of the repository/repositories and accession number(s) can be found below: https://www.ncbi.nlm.nih.gov/genbank/, CP045993.1.

## Author Contributions

GT and AH designed the experiments. GT completed most of the experiments. MM, MN, RR, SM, and AF did a small number of experiments and provided experimental methods. LG carried out the statistical analyses and organized the data. GT and AO created the figures. AO supervised the research design. MH, KY, LG, UP, and AO revised the manuscript. All authors contributed to the article and approved the submitted version.

## Conflict of Interest

The authors declare that the research was conducted in the absence of any commercial or financial relationships that could be construed as a potential conflict of interest.

## Footnotes

1 http://www.r-project.org
