# Supplementary material for: Genomic Insights Into the Antifungal Activity and Plant Growth-Promoting Ability in Bacillus velezensis CMRP 4490
Source: Front Microbiol. 2021 Jan 15;11:618415. doi: 10.3389/fmicb.2020.618415 (PMC7844144; doi:10.3389/fmicb.2020.618415)
Supplement: Supplementary file 1 [file Data_Sheet_1.docx]

**Supplementary Materials**

Genomic Insights Into the Antifungal Activity and Plant Growth-Promoting Ability in *Bacillus velezensis* CMRP 4490

Gustavo Manoel Teixeira^1^, Mirela Mosela^1^, Maria Luiza Abreu Nicoletto^1^, Renan Augusto Ribeiro^2^_,_ Mariangela Hungria^2^, Khamis Youssef^3^, Allan Yukio Higashi^4^, Silas Mian^4^, André Sampaio Ferreira^4^, Leandro Simões Azeredo Gonçalves^4^, Ulisses de Padua Pereira^5^, Admilton Gonçalves de Oliveira^1, 6,^*

^1^ Department of Microbiology, State University of Londrina, Londrina, PR 86057-970, Brazil

^2^Soil Biotechnology Laboratory,Embrapa Soja, Londrina, PR, Brazil

^3^Agricultural Research Center, Plant Pathology Research Institute, 12619 Giza, Egypt

^4^Department of Agronomy, State University of Londrina, Londrina, PR 86057-970, Brazil,

^5^ Department of Preventive Veterinary Medicine, State University of Londrina, Londrina, PR 86057-970, Brazil

^6^ Laboratory of Electron Microscopy and Microanalysis, State University of Londrina, Londrina, PR 86057-970, Brazil

*** Correspondence:**Correspondence: Admilton Gonçalves de Oliveira; admilton@uel.br

Keywords: root colonization ability; biosynthetic gene clusters; soilborne plant pathogens; plant-growth promoting rhizobacteria; biocontrol.

**Table S1.**Genes related to biofilm formation/regulation on *Bacillus velezensis* CMRP 4490.

| Genes | Position on *Bacillus velezensis* CMRP 4490 (bp) | % of identity with reference | Described function on subtwiki[54] |
| --- | --- | --- | --- |
| *abrB* | 252940 a 252651 | 91 % | Repressor of genes that induces sporulation |
| *degQ* | 3241639 a 3241504 | 87% | Stimulates the production of degradative enzymes and extracellularpoli-gama-glutamate |
| *degU* | 3647286 a 3646597 | 86% | Capsule biosynthesis (along with SwrA), DegU non phosphorylated is necessary for motility on swarming |
| *epsA* | 3512467 a 3511760 | 99% | Synthesis of extracellular polysaccharides |
| *epsB* | 3511754 a 3511074 | 99% |  |
| *epsC* | 3510827 a 3509034 | 99% |  |
| *epsD* | 3509018 a 3507879 | 98% |  |
| *epsE* | 3507882 a 3507043 | 98% | Motility and glycosyltransferase inhibitor needed for EPS biosynthesis |
| *epsF* | 3507047 a 3505911 | 99% |  |
| *epsG* | 3505907 a 3504804 | 99% |  |
| *epsH* | 3504785 a 3503748 | 99% |  |
| *epsI* | 3503743 a 3502667 | 99% | Synthesis of poli-N-acetyl glucosamine |
| *epsJ* | 3502670 a 3501636 | 99% |  |
| *epsK* | 3501639 a 3500122 | 99% |  |
| *epsL* | 3500125 a 3499517 | 99% |  |
| *epsM* | 3499520 a 3498873 | 99% |  |
| *epsN* | 3498868 a 3497696 | 99% |  |
| *epsO* | 3497717 a 3496752 | 99% |  |
| *galE1* | 1392187 a 1391195 | 99% | Galactose utilization |
| *kinA* | 1573488 a 1575308 | 78% |  |
| *motA* | 1536204 a 1535389 | 79% |  |
| *motB* | 1535417 a 1534675 | 76% |  |
| *remA* | 1789679 a 1789948 | 97% | Transcriptional regulator of genes from the extracellular matrix, acts alongside with SinR, AbrB and DegU |
| *sigH* | 324256 a 324893 | 88% |  |
| *sigW* | 415480 a 416043 | 83% |  |
| *sinI* | 2676152 a 2676325 | 80% | Biofilm formation control |
| *sinR* | 2676359 a 2676694 | 97% | Transcriptional regulator of post-exponential-phase responses genes |
| *sipW* | 2678176 a 2677592 | 99% | Bifunctional signal peptidase I that controls surface-adhered biofilm formation and processes TasA and TapA |
| *spo0A* | 2642989 a 2642189 | 99% | Regulator of the sporulation initiation |
| *srfAA* | 571928 a 573918 | 79% | Surfactin synthesis |
| *srfAB* | 564380 a 575138 | 75% |  |
| *srfAC* | 575178 a 578974 | 87% |  |
| *swrA* | 3622716 a 3622380 | 84% | Important activator of the flagellar biosynthesis, controls the number of flagellar basal bodies, controls degU activity |
| *swrB* | 1865316 a 1865494 | 82% | Controls SigD activity. Activates type 3 flagellar secretion by the protein membrane FliP |
| *swrC* | 895855 a 898998 | 80% |  |
| *tasA* | 2677527 a 2676742 | 99% | Major component of the biofilm matrix, forms amyloid fibers |
| *yhxB* | 1141855 a 1143597 | 99% |  |
| *yqxM* | 2678819 a 2678148 | 99% |  |

**
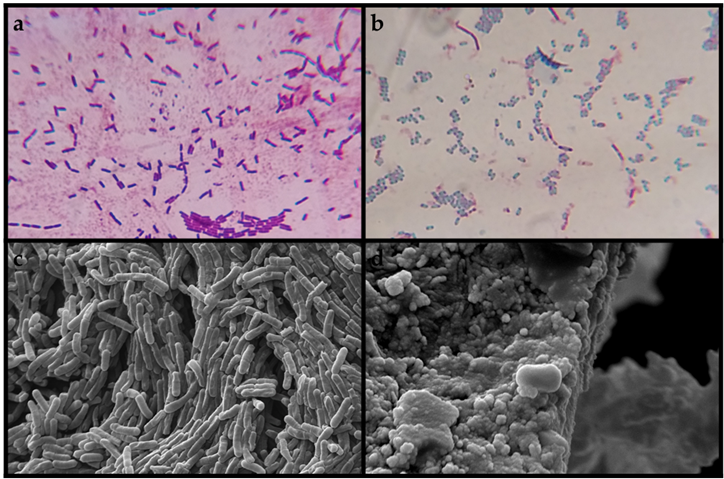
**

Figure S1. Top left: gram staining showing that *Bacillus velezensis* CMRP 4490 is a rod shaped gram-positive bacteria; Top right: Wirtz-Conklin method confirming the strain ability of forming endospores, cells were obtained after 3 days of cultivation at 28 °C; Bottom left: SEM images from the strain’s colony showing its arrangement; Bottom right: SEM image from an endospore formed after 72h of fermentation and then lyophilized.

**
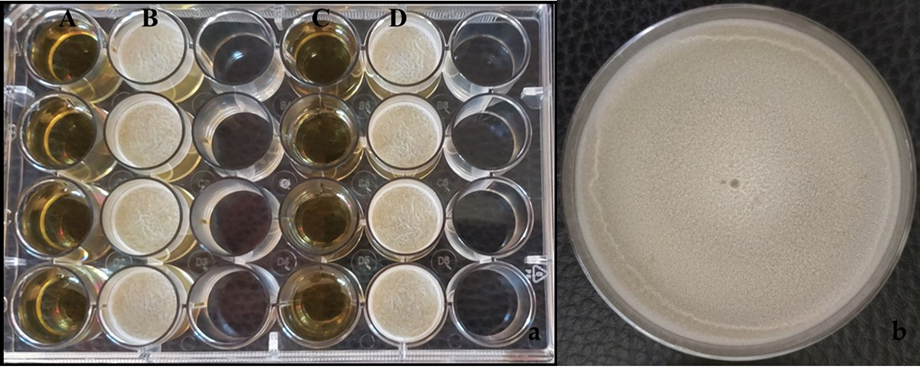
**

**Figure S2:** (a) Pellicle formation on liquid-air interface on static liquid medium. Columns A and C: Medium Control; Columns B and D: inoculated with 10 µL of solution containing *Bacillus velezensis* CMRP 4490 cells adjusted to the tube 0.5 of MacFarland scale. (b) *In vitro* tests made with CMRP 4490, its motility capacity was tested on a TSA 0,8%. A cell inoculum was put in the middle of the dish and its growth was evaluated after 24h. *Bacillus* velezensis CMRP 4490 was able to colonize the whole surface of the medium because of its ability to move thanks to flagella that its genome showed that has the capacity to synt
